# Supplementary material for: Systematic tissue annotations of genomics samples by modeling unstructured metadata
Source: Nat Commun. 2022 Nov 8;13:6736. doi: 10.1038/s41467-022-34435-x (PMC9643451; doi:10.1038/s41467-022-34435-x)
Supplement: Supplementary file 3 — Reporting Summary [file 41467_2022_34435_MOESM3_ESM.pdf]

nature portfolio

Corresponding author(s): Arjun Krishnan

Last updated by author(s): Oct 10, 2022

Reporting Summary

Nature Portfolio wishes to improve the reproducibility of the work that we publish. This form provides structure for consistency and transparency in reporting. For further information on Nature Portfolio policies, see our Editorial Policies and the Editorial Policy Checklist.

Statistics

For all statistical analyses, confirm that the following items are present in the figure legend, table legend, main text, or Methods section.

n/a	Confirmed	
☐	☒	The exact sample size (*n*) for each experimental group/condition, given as a discrete number and unit of measurement
☒	☐	A statement on whether measurements were taken from distinct samples or whether the same sample was measured repeatedly
☐	☒	The statistical test(s) used AND whether they are one- or two-sided<br>*Only common tests should be described solely by name; describe more complex techniques in the Methods section.*
☒	☐	A description of all covariates tested
☐	☒	A description of any assumptions or corrections, such as tests of normality and adjustment for multiple comparisons
☐	☒	A full description of the statistical parameters including central tendency (e.g. means) or other basic estimates (e.g. regression coefficient) AND variation (e.g. standard deviation) or associated estimates of uncertainty (e.g. confidence intervals)
☐	☒	For null hypothesis testing, the test statistic (e.g. *F*, *t*, *r*) with confidence intervals, effect sizes, degrees of freedom and *P* value noted<br>*Give P values as exact values whenever suitable.*
☒	☐	For Bayesian analysis, information on the choice of priors and Markov chain Monte Carlo settings
☒	☐	For hierarchical and complex designs, identification of the appropriate level for tests and full reporting of outcomes
☐	☒	Estimates of effect sizes (e.g. Cohen's *d*, Pearson's *r*), indicating how they were calculated

Our web collection on statistics for biologists contains articles on many of the points above.

Software and code

Policy information about availability of computer code

Data collection

Data was downloaded from ArrayExpress using the curl command. Code used to process the data are available at https://github.com/krishnanlab/txt2onto

Data analysis

We have made the trained NLP-ML models, a Python utility for text-based tissue classification, and demo scripts at https://github.com/krishnanlab/txt2onto, along with extensive documentation. Given an input file where each line is a piece of text to be classified, the txt2onto utility will perform the necessary text preprocessing, create an embedding for each piece of text, and then run each embedding through our pre-trained tissue models. The repository also includes a set of utilities for training new NLP-ML models for a user-defined problem.

R 3.3
affy 1.52.0
frma 1.26.0

Python 2 for TAGGER and MetaSRA
Python 3.7.7 for NLP-ML
Flair 0.8.0
NLTK 3.6.2
Scikit-Learn 0.20.3

For manuscripts utilizing custom algorithms or software that are central to the research but not yet described in published literature, software must be made available to editors and reviewers. We strongly encourage code deposition in a community repository (e.g. GitHub). See the Nature Portfolio guidelines for submitting code & software for further information.

Data

Policy information about availability of data

All manuscripts must include a data availability statement. This statement should provide the following information, where applicable:

- Accession codes, unique identifiers, or web links for publicly available datasets
- A description of any restrictions on data availability
- For clinical datasets or third party data, please ensure that the statement adheres to our policy

All the gold standard annotations (labeled examples) used to train all the models and the cross-validation splits used to evaluate the models are available in the repository https://github.com/krishnanlab/txt2onto. Lists of samples from specific microrray platforms (available at https://github.com/krishnanlab/txt2onto/tree/main/gold_standard) were downloaded from GEOMetaDB https://doi.org/doi:10.18129/B9.bioc.GEOmetadb. Metadata were downloaded from ArrayExpress https://www.ebi.ac.uk/arrayexpress/. Source data files necessary to recreate our figures are provided in the repository and with this paper.

Field-specific reporting

Please select the one below that is the best fit for your research. If you are not sure, read the appropriate sections before making your selection.

[x] Life sciences [ ] Behavioural & social sciences [ ] Ecological, evolutionary & environmental sciences

For a reference copy of the document with all sections, see nature.com/documents/nr-reporting-summary-flat.pdf

Life sciences study design

All studies must disclose on these points even when the disclosure is negative.

Sample size	In this study, the number of samples for specific tests equals the number of tissues for which there were sufficient number of training samples based on manual curation and therefore only determined based on the availability of data. No further selections were made.
Data exclusions	No data exclusions were made after generating the gold-standard based on manual curation.
Replication	The multiple variable in this study (i.e. various tissues) are independent instances and not replicates of each other without any notion of replicate measurements.
Randomization	Difference between methods were tested based on all tissues and by partitioning tissues into subgroups based on the amount of training data. Otherwise, randomization is not relevant here.
Blinding	All data were directly used to test the differences between methods. Since the data (i.e. from different tissues) were split into groups and compared to each other, blinding is not necessary here.

Reporting for specific materials, systems and methods

We require information from authors about some types of materials, experimental systems and methods used in many studies. Here, indicate whether each material, system or method listed is relevant to your study. If you are not sure if a list item applies to your research, read the appropriate section before selecting a response.

Materials & experimental systems

n/a	Involved in the study	
[x]	[ ]	Antibodies
[x]	[ ]	Eukaryotic cell lines
[x]	[ ]	Palaeontology and archaeology
[x]	[ ]	Animals and other organisms
[x]	[ ]	Human research participants
[x]	[ ]	Clinical data
[x]	[ ]	Dual use research of concern

Methods

n/a	Involved in the study	
[x]	[ ]	ChIP-seq
[x]	[ ]	Flow cytometry
[x]	[ ]	MRI-based neuroimaging
